# Percolation Threshold of Bacterial Nanocrystals in Biopolymeric Matrices to Build Up Strengthened Biobased Food Packaging

**DOI:** 10.3390/foods14071123

**Published:** 2025-03-24

**Authors:** Fabíola Medeiros da Costa, Pamela Thais Sousa Melo, Pedro Henrique Kenzo Nishimoto, Marcos Vinicius Lorevice, Fauze Ahmad Aouada, Márcia Regina de Moura

**Affiliations:** 1 Hybrid Composites and Nanocomposites Group (GCNH), Department of Physics and Chemistry, School of Engineering, São Paulo State University (UNESP), Ilha Solteira 15385-000, SP, Brazil; medeiros.costa@unesp.br (F.M.d.C.); pamelathais_@hotmail.com (P.T.S.M.); fauze.aouada@unesp.br (F.A.A.); 2 Brazilian Nanotechnology National Laboratory (LNNano), Brazilian Center for Research in Energy and Materials (CNPEM), Campinas 13083-970, SP, Brazil; pedro.nishimoto@gmail.com (P.H.K.N.); marcos.lorevice@lnnano.cnpem.br (M.V.L.)

**Keywords:** gelatin, pectin, HPMC, bacterial cellulose nanocrystals, percolation threshold, packaging

## Abstract

Bacterial cellulose nanocrystals (BCNCs) extracted from cellulose residues, resulting from film-cutting operations used for the commercial production of dressings, were studied as reinforcement for films based on gelatin, pectin, and hydroxypropylmethyl cellulose (HPMC). The biopolymer matrices differ in their monomer and functional group (gelatin: -COOH and -NH; pectin: -COOH and HPMC -OH). The addition of BCNCs into a polymer matrix for biopolymeric nanocomposite formulation was based on values around the theoretical percolation threshold. The results of this study showed that the BCNCs had a diameter and mean length range of (27 ± 1) nm and (180 ± 10) nm, respectively, producing films reaching 120.13 MPa of tensile strength, 10.9 GPa of Young’s modulus, and a toughness of 335.17 × 10^6^ J/m^3^. All films showed good transparency and a smooth surface. Surface micrographs (SEM) revealed homogeneous, compact, smooth regions, and no macropores. The crystallinity index of the BCNCs produced was 68.69%. The crystallinity of the gelatin, pectin, and HPMC films improved from 10.25 to 44.61%, from 29.79 to 53.04%, and from 18.81 to 39.88%, respectively. These results show the possibility of using films for freeze-dried food packaging.

## 1. Introduction

The development of new packaging from biopolymers can be a viable alternative to meet the new demands of the “micro-plastic free” market (European Commission, 25 September 2023 [1]), being also in line with the goals proposed by the 2030 agenda for sustainable development [2,3,4,5,6]. These biopolymers can be used as raw materials in the production of food packaging due to their wide range of functionalities [7,8]. Another interesting point for their use is their low toxicity [4,5,9,10].

Bioplastics are materials based on biopolymers such as starch, proteins, cellulose, and other polysaccharides, which are considered safe for consumption (FDA [11]; GRAS Notice No. GRN 000213, 2007 [12]). Among the materials used to produce bioplastics, gelatin, pectin, and hydroxypropylmethyl cellulose (HPMC) are strong candidates for manufacturing new packaging.

As a primary component, gelatin has a noteworthy potential to form edible and/or biodegradable films, being also abundant and relatively low-cost. It is a high molecular mass polypeptide (80 to 125 kDa) composed of amino acids, mainly glycine (27%), followed by hydroxyproline and proline (25%) [13]. The three-dimensional triple helix structure of the gelatin protein provides physical resistance; moreover, the different amino acids present in its structure absorb UV radiation and, if applied as films or coatings for foods, can protect them from oxidative damage caused by exposure to UV radiation [14]. Although gelatin-based films can be sufficiently rigid, they tend to be brittle, that is, they are a rigid material with low elongation at break (<25%) [14].

Pectin, a soluble biopolymer composed of heteropolysaccharides and soluble in water, has been studied for application in the environmentally friendly bioplastic manufacturing process [15]. This soluble macromolecule is composed mainly of D-galacturonic acid β-(1-4) linked to a significant amount of galactose, arabinose, and rhamnose, in addition to having excellent gelling properties [16]. Furthermore, it is an anionic, amorphous, and non-toxic biopolymer [16]. Given its properties, pectin is widely used to produce biopolymeric nanocomposites with potential applications in active packaging [16].

Another raw material used in the production of bioplastics is hydroxypropylmethyl cellulose (HPMC). HPMC preparation relies on replacing the hydroxyl group with hydroxypropyl and methyl groups in the anhydroglucose backbone. That molecular trait gives the structure several degrees of substitution [17]. It is a biopolymer derived from cellulose [18] with wide application in the food and pharmaceutical industries since it is considered safe for human consumption. Due to its good film-forming properties, it is a common raw material in film-coated tablet formulations [19]. As an excipient, HPMC can be used as a thickener, binder, solubility enhancer, etc.

Biopolymers usually have hydrophilic intrinsic characteristics, which make them highly water-soluble materials. However, they have inferior mechanical properties when compared to conventional polymers. Thus, applying biopolymer films in situations that require mechanical resistance and a water vapor barrier still lacks viability [14]. In this context, nanotechnology, with the effective use of reinforcement materials, tends to enhance food packaging technology to develop nanocomposites as films with advanced mechanical and barrier properties [7].

Nanocellulose, widely used as reinforcement in biopolymeric matrices, can be classified into cellulose nanocrystals, cellulose nanofibrils, and bacterial cellulose (BCNCs) [20,21]. BCNCs have shown promise in enhancing new packaging due to their properties, such as flexibility, high degree of crystallinity, high surface area, high elastic modulus, large water retention capacity, low density [22], and physiologically inert properties [20,23]. BCNCs have a needle-shaped morphology, and their higher aspect ratio and low density make them good candidates for reinforcement in the polymer matrix due to the ability to form a percolated network [20,21,24]. Although cellulose nanocrystals’ high surface area and hydrogen bonding effect (three OH groups per anhydroglucose unit) undermine their homogenous dispersion in a hydrophobic matrix [25,26,27].

Even with reduced cellulose nanocrystal loading, excellent mechanical properties can be obtained in a polymer matrix when adding nanostructures. Such properties originate from the high rigidity of the crystalline zones of cellulose [27]. Suitable formulations enable a network of nanocrystals, allowing mechanical percolation through a polymer matrix. The formation of this network is conditioned by the homogeneous dispersion of the charge, in addition to the percolation threshold, which depends on the proportion of nanocrystals and the charge/charge Coulombic interactions [21]. A uniform dispersion of nanoparticles throughout a polymer matrix results in a large interfacial area, which alters the molecular mobility, relaxation behavior, and resulting mechanical properties [28].

In this context, this study dives into the extraction of nanocrystals from bacterial cellulose and the production and characterization of biopolymeric nanocomposites based on gelatin, pectin, and HPMC nanostructured with BCNCs. It is worth mentioning that the biopolymer matrices differ in their monomer and functional group (gelatin: -COOH and -NH; pectin: -COOH and HPMC -OH). Gelatin is a cationic polymer; pectin is anionic; and HPMC is neutral. The BCNCs suspensions were obtained by acid hydrolysis of bacterial cellulose (by-products) from samples of dressing residues and introduced into the biopolymer matrices. This study focused on the impact of the behavior of BCNCs, with fractions around the percolation threshold, on the final mechanical properties and their compatibility with the matrix to optimize the stress distribution across the multifunctional film according to the percolation threshold. The main hurdle of using BCNCs is derived from their homogeneous dispersion within a polymer matrix due to cellulose nanocrystals’ strong tendency to self-associate, triggered by the ubiquity of interacting surface hydroxyl groups. This property is desirable for forming load-bearing percolation architectures within the host polymer matrix. However, these interparticle interactions can cause aggregation during the preparation of the biopolymeric nanocomposite and limit the potential for mechanical reinforcement. This phenomenon is amplified when the particle size decreases [21].

Morphological characterization and spectroscopic analyses of the BCNCs and biopolymeric nanocomposites films were performed to support the mentioned correlations.

## 2. Materials and Methods

The reagents used in this work were not further purified. Gelatin (CAS 9000-70-8) was purchased from Dinâmica Química Contemporânea (São Paulo, Brazil). HPMC Methocel^®^ K4M (CAS 9004-65-3, average methoxyl/hydroxypropyl ratio: 2.26) was obtained through a donation by Danisco Brasil Ltd.a^®^, pectin (CAS 9000-69-5, methoxylation degree above 50%) was kindly donated by CP Kelco© (Limeira, Brazil), sulfuric acid (CAS 7664-93-9, concentration 64%) was purchased from Êxodo Científica (São Paulo, Brazil) and potassium bromide (CAS 7758-02-3) was purchased from Sigma-Aldrich (USA). Dialysis membrane, Servapor Dialysis tubing MWCO 12000-14, was purchased from Serva (São Paulo, Brazil). Bacterial cellulose residues, resulting from film-cutting operations used for the commercial production of dressings, were provided by Nexfill^®^ (Brazil) and used to obtain BCNCs. Deionized Milli-Q water (Millipore Corp., St. Louis, MI, USA; resistivity of 18.2 MΩ) was used in all analyses.

### 2.1. Extraction of Bacterial Cellulose Nanocrystals (BCNCs)

The extraction method used was acid hydrolysis, as described by Melo et al. [29]. Dry films of bacterial cellulose (BC), obtained as waste from the supplier company, were mechanically crushed in a blender. The powdered fibers were hydrolyzed in 64% *v*/*v* sulfuric acid at 50 °C under constant mechanical stirring for 50 min. The amount of acid used was 17.5 mL per gram of BC. The hydrolysis reaction stopped by diluting the mixture 10 times with ice-cold water. The suspension was centrifuged at 600 rpm for 10 min. After discarding the supernatant, the precipitate was placed in a MWCO 12000-14 dialysis membrane tube and then submerged in milli-Q water until reaching a pH of approximately 6. Thus, the BCNCs content was determined by measuring the total solids obtained after drying in an oven at 70 °C for 24 h.

### 2.2. Topographic Characterization of Cellulose Nanocrystals

The BCNCs were dispersed at 0.001 wt. % in ultrapure water using a Branson Model 250 sonicator and then deposited onto a mica substrate. Atomic force microscopy (AFM) images were acquired using a Bruker Multimode 8 equipped with Nanoscope V electronics, operating in PeakForce Tapping mode. The scan rate was set to 1 Hz, with a probe spring constant of 0.4 N/m and a resonance frequency of 70 kHz. The images were processed and analyzed using Gwyddion v. 2.53 software. The size particle distribution was determined by measuring 100 randomly selected particles in diverse regions of three different AFM images.

### 2.3. Formulation, Preparation, and Characterization of Nanocomposite Films

The addition of BCNCs into a polymeric matrix for biopolymeric nanocomposite formulation was based on values around the theoretical percolation threshold, calculated according to the semiempirical Halpin–Tsai model [30]. This model assumes a homogeneous distribution of the nanofiller, an ideal interaction between the nanofiller and the matrix, and no interaction between the nanofillers. From this, it is possible to estimate the mechanical properties of a composite as a function of the nanofiller percolation processes, given the properties and volume fractions of the matrix and nanofiller [31,32]. First, the volume aspect ratio (*V_RC_*) of the BCNCs was calculated according to Equation (1):

(1) $$V_{RC}=0.7\times (\frac{D}{L})$$

where *L* and *D* are the average length and diameter of the nanocrystals, respectively. Thus, the *V_RC_* found was ~10.5% volume for the BCNCs. With the density of the BCNCs, 1.6 g/cm^3^ [27], the *V_RC_* was converted to percentage values (%) in mass, and the value found was 16.8% mass. Considering the densities of the matrices—gelatin (1.2 g/cm^3^), pectin (1.35 g/cm^3^), and HPMC (1.39 g/cm^3^)—the BCNCs contents both above and below the percolation threshold were determined as 14 wt. % for gelatin, 12.4 wt. % for pectin, and 12 wt. % for HPMC. Therefore, the concentration of biopolymers added in the matrices was based on average values from previous studies by the GCNH group, with variations of ±5 %. Table 1 shows the composition of the films and their respective acronyms. In all cases, the percentage of biopolymer was fixed at 2.0 wt. %, and the dry mass of BCNCs was calculated from the dry mass of the matrix.

**Figure 1:**
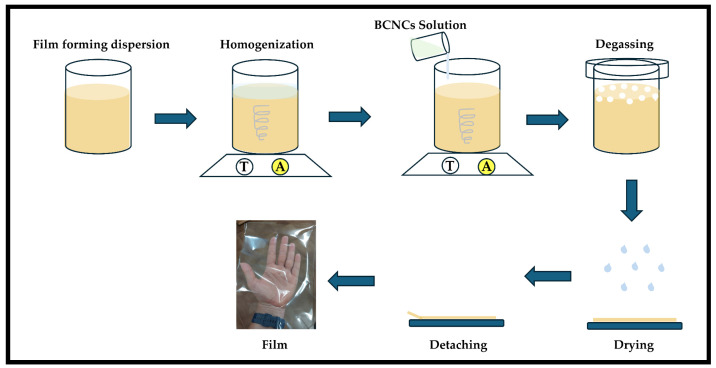
Simplified scheme highlighting film production by casting.

Preparing film-forming suspension demanded two stages (Figure 1). The solubilization of biopolymers in aqueous suspension represented the first, where known amounts of pectin and HPMC were suspended in water and subjected to magnetic stirring for 6 and 24 h, respectively, until the biopolymers were completely dissolved. Gelatin, on the other hand, was first swollen for 1 h in water and then heated in a water bath up to 50 °C. After reaching this temperature, the solution was placed under magnetic stirring for 10 min and then cooled to 30 °C. Subsequently, the two dispersions were mixed—the biopolymer and the BCNCs—and the latter was added to the film-forming suspensions and stirred for 30 min.

All film-forming suspensions containing BCNCs were degassed and poured onto a support (polyethylene terephthalate) measuring 20 × 20 cm (Figure 1) and left to dry completely at room temperature (casting method). Prior to characterization, the films were removed from the support and equilibrated at a relative humidity of 50% for at least 48 h.

### 2.4. Scanning Electron Microscopy (SEM)

The surface morphology of the liquid nitrogen freeze-fractured cross-section of the films was analyzed using an EVO LS15 Scanning Electron Microscope (Zeiss, Jena, Germany) equipped with an EDS (energy dispersive spectrum) detection system. Prior to analysis, the test samples were coated with a thin layer of gold/palladium alloy through sputtering using a Quorum Q150T E. Magnifications ranging from 1000 to 10,000× and an accelerating voltage of 10 kV were used for imaging.

### 2.5. X-Ray Diffractometry (XRD)

XRD measurements of the BCNCs and biopolymeric nanocomposites were performed on a DRX-6000 diffractometer (Shimadzu, Japan) using CuKα radiation (λ = 1.54056 Å), with an angular range of 5–50° and a step size of 2°·min^−1^. The crystallinity index (*Ci*) was determined from the results obtained by applying Equation (2) [25,33]:

(2) $$Ci=\frac{\Sigma A_{Crystal}}{A_{Total}}\times 100\%$$

where *A_Total_* is the total area under all diffraction, and *ΣA_Crystal_* is the sum of the areas corresponding to the crystalline peaks.

### 2.6. Mechanical Properties: Tensile Strength, Young’s Modulus, Deformation, and Toughness

First, the thickness of the films was measured using a digital micrometer (No. 7326, Mitutoyo Corporation, Kanagawa, Japan) at five random positions around each film. The mechanical properties, tensile strength (MPa), Young’s modulus (GPa), yield stress (MPa), elongation at break (%), and toughness (J/m^3^) of the films were determined from the stress–strain curve resulting from the tensile test. The films were cut into width and length of 5 mm and 22 mm, respectively, and each sample was tested at least 9 times at a temperature of 25 °C and a relative humidity of 50%. The test was performed on a universal testing machine (model 3369, Instron Corp., Canton, MA, USA), operating with a 50 N load cell and a 10 mm·min^−1^ deformation rate, according to ASTM International D1708-18 standard [34]. All samples were left in a desiccator for 48 h before testing to control relative humidity at around 50% with silica gel pellets.

The point of utmost tensile stress value represented the film’s tensile strength. The determination of Young’s modulus represented by the slope of the curve in the elastic region (0–2% deformation) and the stress at the yield point followed the ASTM International D638-14 standard [35]. The deformation was determined by the values of the abscissa axis, while the toughness value was acquired from the total area of the stress–strain curve. Tensile strength, Young’s modulus, and deformation values respected the Instron Universal Testing Model 3369.

### 2.7. Statistical Analysis

One-way analysis of variance (ANOVA) was used to compare more than two data sets using Origin software, version 6.0 (Origin Lab, Northampton, MA, USA). All data were represented as mean ± standard deviation. Significant differences (*p* ≤ 0.05) are denoted by showing the data in tables with different letters.

After performing a multiple regression analysis for the experimental data of mechanical properties, the quadratic model was selected. Subsequently, a final model equation for predicting the response of mechanical properties in relation to matrices and percentage reinforcement (BCNCs) was obtained using Statistica software, version 7.0, Tulsa, OK, USA.

## 3. Results and Discussion

### 3.1. Characterization of Nanostructures

**Figure 2:**
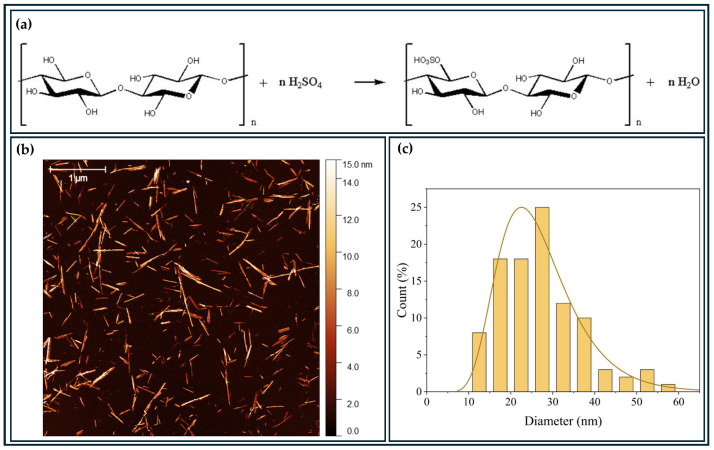
AFM analysis of BCNCs (**b**,**c**) obtained from acid hydrolysis (**a**).

The BCNCs were obtained by acid hydrolysis using H_2_SO_4_ (Figure 2a). The solid content of the BCNCs solution after hydrolysis was 1.62 wt.%. This production of nanocrystals was based on the work carried out by our group [29], in which the BCNCs production resulted in negatively charged nanostructures due to the binding of sulfate groups originating from sulfuric acid through the hydrolysis reaction.

Regarding the morphology and dimensions of the BCNCs (Figure 2b,c), they exhibited an average diameter of (27 ± 1) nm and an average length of (180 ± 10) nm, yielding an average aspect ratio (*L*/*D*) of (6.67 ± 0.3). These length and average diameter values were consistent with those reported in the literature [36]. Overall, the structures displayed a needle shape, which is characteristic of the acid hydrolysis method [36,37].

### 3.2. Film Characterization

#### 3.2.1. External Visual Appearance

**Figure 3:**
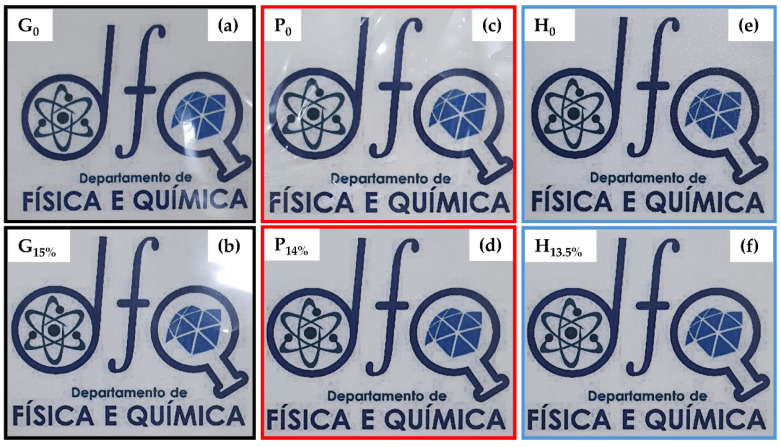
Photographs of films based on gelatin (**a**,**b**), pectin (**c**,**d**), HPMC (**e**,**f**) pure, and incorporated with different BCNCs contents.

All films showed good transparency and a smooth surface (Figure 3a–f), suggesting BCNCs did not affect the color and homogeneity of biopolymer nanocomposites. Even with a high BCNCs content, these films remained cohesive and transparent. That is essential, as aggregates of nanoparticles could impair light transmission. Furthermore, film brightness and transparency have a noticeable impact on the appearance of packaged foods [38].

#### 3.2.2. Scanning Electron Microscopy (SEM)

The film’s final structure is influenced by the interactions of its components and its dispersion drying conditions. This structure significantly affects film properties. In this sense, the microstructural analysis of films reveals relevant information concerning the arrangement of the components [39].

**Figure 4:**
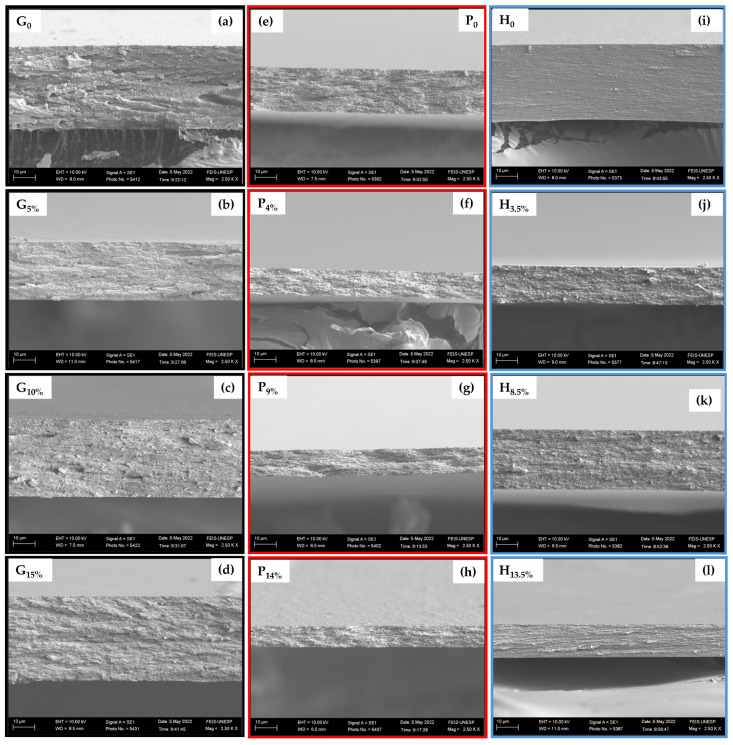
Cross-sectional microscopies of cryogenic fractures of gelatin (**a**–**d**), pectin (**e**–**h**), and HPMC (**i**–**l**) films incorporated with different amounts of bacterial cellulose nanocrystals (magnification 2500×; bar = 10 µm).

Figure 4a–l shows the surface micrographs of the cross-section of the samples in the cryogenic fracture. The micrographs present the morphologies of the films formed by gelatin, pectin, and HPMC with varied BCNCs contents in each matrix. Moreover, the regions are homogeneous, compact, smooth, without macropores, and without residual points, that is, fracture-forming points. That indicates that the BCNCs had homogenous dispersion in the biopolymeric substrates during the film formation process, and there was no phase separation, suggesting high compatibility between the film components. That may be attributed to the abundant free hydroxyl groups of BCNCs, which interacted with the biopolymer chains and behaved as a reinforcing agent in the matrix [40].

#### 3.2.3. X-Ray Diffractometry (XRD)

**Figure 5:**
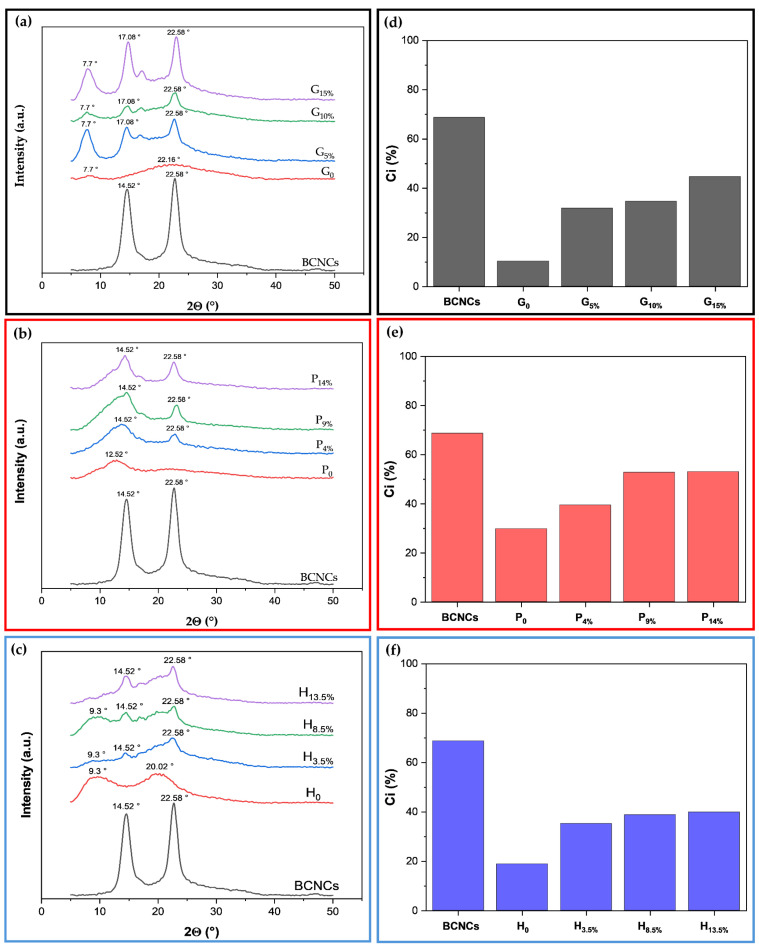
X-ray diffractograms of films (**a**–**c**) and crystallinity index (**d**–**f**) of gelatin (G), pectin (P), and HPMC (H) incorporated with different amounts of bacterial cellulose nanocrystals (BCNCs).

Crystallinity is a relevant factor specifically influencing the mechanical properties of materials. Figure 5a–c and Figure 5d,e display the X-ray diffractograms and crystallinity indices of BCNCs and films, respectively. The BCNCs (Figure 5a–c) exhibited peak intensity at the Bragg angle 2Θ of approximately 14.52° and 22.58°. These peaks correspond to the lattice planes (10) and (200), respectively, which are characteristic of type I cellulose [5,25,41].

The degree of crystallinity of BCNCs can increase as the amorphous portions undergo acid hydrolysis [29]. In this process, hydronium ions penetrate the amorphous zones, promoting the hydrolytic cleavage of glycosidic bonds and releasing individual crystallites, while the crystalline zones are more resistant to chemical attack [5,21]. The crystallinity index of the BCNCs produced was 68.69%. Nascimento et al. [42] reported that the spatial arrangement of bacterial cellulose provides high crystallinity, up to 80−90%, significantly higher than the 40−60% associated with plant-derived cellulose. Vasconcelos et al. [36] extracted BCNCs in acid hydrolysis, varying the H_2_SO_4_ concentration and hydrolysis time under conditions similar to those used in this study. However, the crystallinity index of the BCNCs they obtained was only 22%, whereas the optimal conditions they identified were at an acid concentration of 50% (*v*/*v*) for 60 and 120 min, yielding crystallinity indices of 91 and 92%, respectively. Therefore, the difference in the crystallinity index may be due to the hydrolysis conditions used during the extraction of the BCNCs, as more severe conditions may result in a change in the orientation of the cellulose chains [5,36].

The X-ray diffraction patterns presented in Figure 5a reflect the structural variation in aggregation of the gelatin chains in the different films. The XRD of the gelatin film has two diffraction peaks at 2θ ≈ 7.7° and 2θ ≈ 22.16°, representing collagen as a triple helix structure and the amorphous phase of free single helix chains, respectively, indicating partial crystallization of the biopolymer (10.25%) [43]. This result is consistent with what has already been reported in the literature [44]. With the addition of BCNCs (Figure 5a), the 7.7° 2θ peak of gelatin intensified, indicating an increase in the crystallinity of the biopolymeric nanocomposite compared to the pure biopolymer. That is evident from the crystalline peaks at 2θ values of 7.7°, 17.08°, and 22.58°, and the increase in the crystallinity index for the G_5%_, G_10%_, and G_15%_ biopolymeric nanocomposites, which were 31.75%, 34.56%, and 44.61%, respectively (Figure 5d).

As observed for gelatin films, the incorporation of BCNCs into the pectin matrix considerably increased the crystallinity index (Figure 5b,f). The XRD spectrum suggests that the P_0_ film has an amorphous structure, according to the broad and flat peak presented in the diffractogram [44,45]. With the addition of BCNCs, the pectin nanocomposite films presented an intense peak at 2θ of 22.58°, which can be attributed to the BCNCs. However, the crystalline portion at P_0_ (2θ of 12.52°) decreased, indicating that the BCNCs are embedded within the relatively amorphous pectin matrix. This embedding is further supported by the increase in the crystallinity percentage of the biopolymeric nanocomposite films, which rose from just over 20% (P_0_) to 39.44% (P_4%_), 52.75% (P_9%_), and 53.04% (P_14%_) [46].

Figure 5c illustrates the XRD patterns of different HPMC-based biopolymeric nanocomposite films. The semicrystalline structure of HPMC indicated by the peak at 2θ equal to 9.3° and 20.02°, and the crystallinity index was 18.81% (Figure 5f), which is consistent with previous works [47]. In the biopolymeric nanocomposite films, H_3.5%_, H_8.5%_, and H_13.5%_, the peaks were at 2θ = 14.52° and 22.58°, originating from the incorporated BCNCs, since the broadened peak at 2θ of 9.3° in HPMC is usually associated with the semicrystalline regions. The incorporation of BCNCs promoted crystallinity indices of 35.19% (H_3.5%_), 38.79% (H_8.5%_), and 39.88% (H_13.5%_).

### 3.3. Thickness and Mechanical Behavior

The film thickness ranged from 0.015 to 0.041 mm (Table A1, Appendix A). The gelatin-based films had an average value of 0.034 mm; the pectin-based films had an average value of 0.019 mm; and the HPMC-based films had an average value of 0.032 mm.

**Figure 6:**
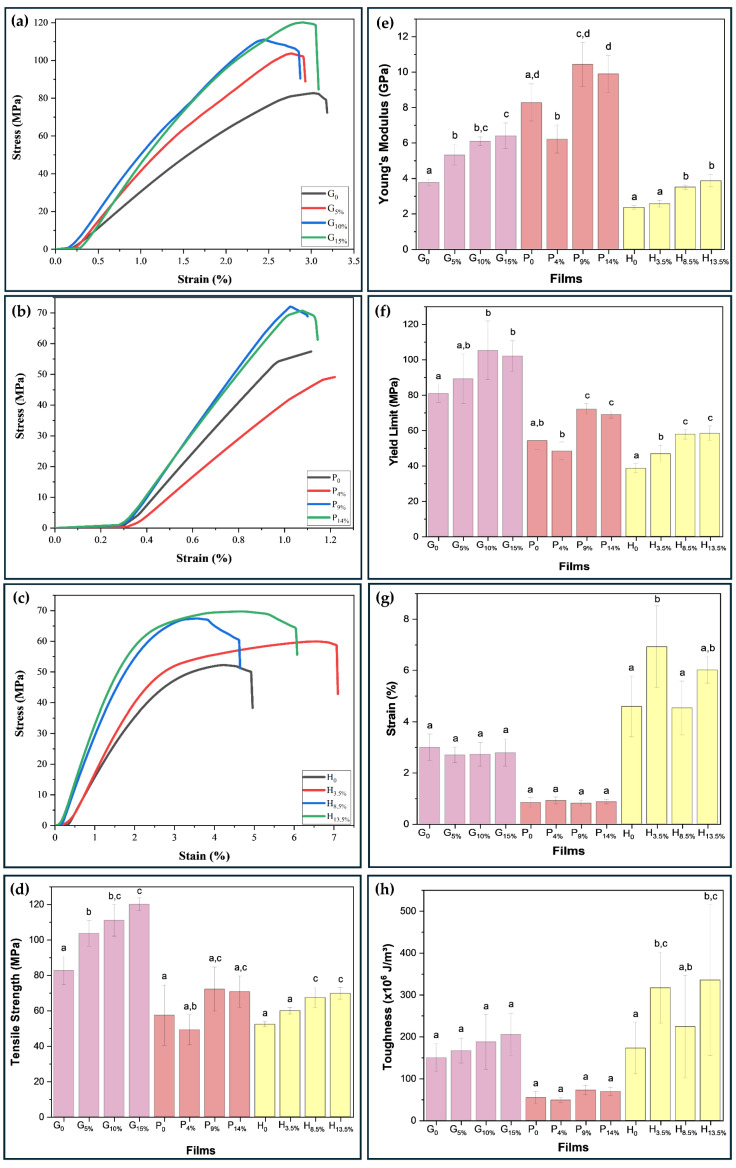
Stress vs. strain curves (**a**–**c**), and mechanical properties (**d**–**h**) (tensile strength (**d**), Young’s modulus (**e**), yield limit (**f**), elongation at break (**g**), and toughness (**h**)) of pure and biopolymeric nanocomposite films. Different letters indicate a significant difference (*p* < 0.05) by the Tukey’s b test.

The mechanical properties of the gelatin-based films (G_0_, G_5%_, G_10%_, and G_15%_), pectin (P_0_, P_4%_, P_9%_, and P_14%_), and HPMC (H_0_, H_3.5%_, H_8.5%_, and H_13.5%_) were investigated. According to Yang et al. [33], mechanical strength and adequate extensibility are basic requirements of a film to withstand external stress and maintain integrity, especially if intended for use in packaging applications. The results are shown in Figure 6a–h. The tensile strength, deformation, Young’s modulus, yield strength, and toughness properties were obtained from the stress–strain curves during the tensile test.

The highest tensile strength value for the gelatin matrix stood out for the biopolymeric nanocomposite loaded with 15% *w*/*w* BCNCs, which increased from 82.66 MPa (G_0_) to 120.13 MPa. The higher crystallinity and degree of orientation of the BCNCs in the films contribute to the higher tensile strength values associated with gelatin films [13], a pattern illustrated in Figure 5a. For the pectin matrix, the biopolymeric nanocomposite containing 9% BCNCs had the highest increase, from 57.44 MPa (P_0_) to 72.13 MPa (P_9%_). This increase is related to a high-strength interface that can efficiently transfer stress between the matrix and the reinforcing agents (e.g., BCNCs) [33]) with high crystallinity (Figure 5a,d). For HPMC-based nanocomposites, the film incorporating 13.5 wt.% BCNCs showed an increase of 17.47 MPa, representing a ~33% improvement.

In general, the biopolymeric nanocomposites presented low elongation at break values, with values for the gelatin, pectin, and HPMC matrix of 2.80, 0.85, and 5.51%, respectively. The possible reason for the high tensile strength with low BCNCs content in the film is due to the hydrogen bonding interactions in the matrix/BCNCs biopolymeric nanocomposites [27] and the fact that BCNCs occupy the free volume spaces within the polymer network of the film-forming matrix, limiting the stretching capacity and mobility of these chains [40].

When reinforced with 15 wt.% BCNCs in the gelatin matrix, Young’s modulus increased by ~70%, ranging from 3.75 to 6.39 GPa. When adding 9 wt.% of BCNCs in pectin film, the increment was ~23%, with values ranging from 8.27 to 10.43 GPa. Regarding HPMC loaded with 13.5 wt.%, the improvement reached ~65%, with values between 2.34 and 3.85 GPa. High values of Young’s modulus, a property that represents the rigidity of the material, can be the result of both strong intermolecular interactions between the matrix and the reinforcement, as well as the homogeneous dispersion of BCNCs in the biopolymer matrix, which provides good mechanical stability for the biopolymeric nanocomposite [48].

The yield stress was used to determine whether there was percolation of BCNCs in the biopolymer matrices. For film G_0_, the yield stress obtained was 81.00 MPa. From 5% to 15% wt. of BCNCs, there was an increase of 5% to 28%, reaching values above 89.24 MPa. For film P_0_, the yield stress found was 54.24 MPa. When reinforcing the pectin film with 4 wt.% of BCNCs, the stress value decreased by ~10.81%. However, when 9 wt.% and 14 wt.% of BCNCs were incorporated, the yield stress of pectin nanocomposite increased by ~32.97% and ~27.19%, respectively, indicating a reinforcement limit for pectin-based films. For the H_0_ film, the yield stress value was 38.59 MPa. When reinforcing it with 3.5 wt.% BCNCs, the yield stress increased by ~21.49%, and with 13.5 wt.% BCNCs, it increased by ~51.43%.

These excellent mechanical improvements may be attributed to the rigid shape of the nanocrystal, and its elementary crystalline form gives rise to remarkable mechanical properties with an axial Young’s modulus in the range of 110–180 GPa and strength around 2–3 GPa [49]. In addition, anisotropic particles, such as cellulose nanocrystals, allow for decreasing the percolation threshold, which is of great importance in providing an interface with mechanical rigidity to avoid aggregation of the nanoparticles [50]. These excellent mechanical properties of cellulose nanocrystals justify the focus on their incorporation into a biopolymer to reinforce the mechanical properties of the material.

Furthermore, the improvement in the mechanical properties may be related to the percolation behavior of the fillers when dispersed throughout the polymer matrices [15,51]. Moreover, the stiffness of the percolated cellulose nanocrystal increases with the aspect ratio of the nanocrystals. That means using cellulose nanocrystals with a higher aspect ratio is more interesting from a mechanical point of view because it first induces a decrease in the critical percolation threshold, and stiffens the continuous network formed. Such a pattern could be attributed to stronger hydrogen bonding between nanocrystals of the high aspect ratio.

Interestingly, under the conditions abovementioned, the host polymer matrix does not play any role in the mechanical stiffness of the material. It corresponds to the greatest mechanical reinforcement effect obtained from these nanostructures. However, many parameters can affect this phenomenon. When inhibiting the formation of this network of percolated nanoparticles, only the high rigidity of crystalline cellulose, nanoscale dimensions, high aspect ratio, dispersion of the nanoparticles, and filler/matrix interactions are involved in the reinforcement phenomenon [21].

**Figure 7:**
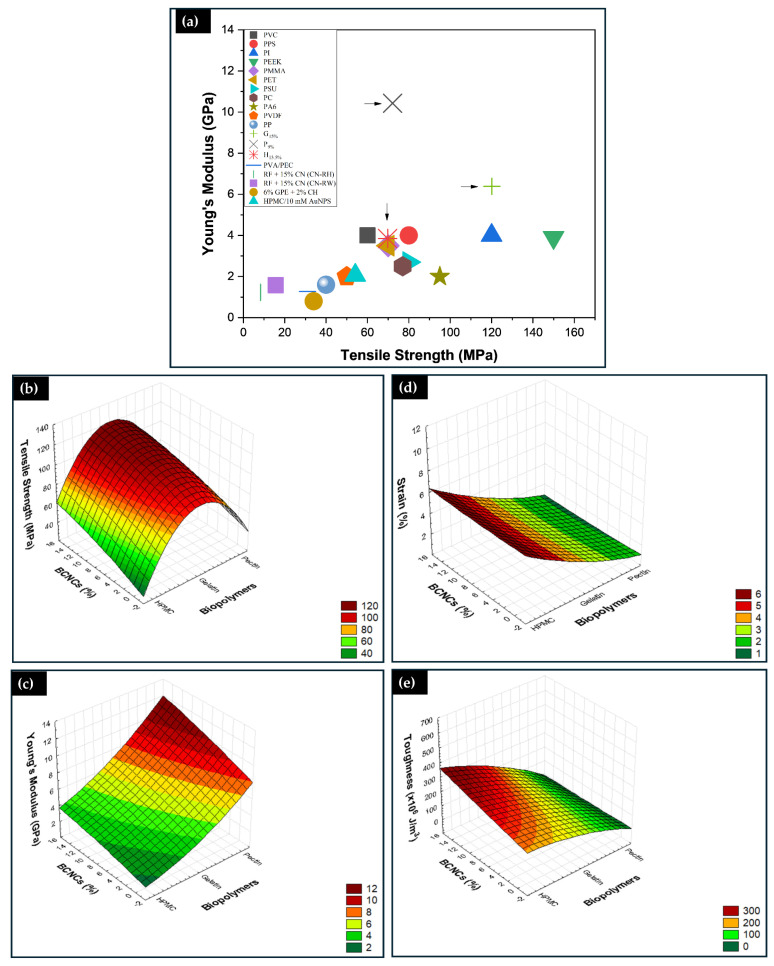
Comparison of Young’s modulus and tensile strength between common petroleum-based synthetic polymers and biopolymers synthesized in other works (**a**), the arrows show the film’s valeus studied in this work; 3D surface plots of significant interaction factors and response variables, showing the effect of mechanical properties across matrices and reinforcement percentages (**b**–**e**).

Figure 7a illustrates Young’s modulus and tensile strength of one biopolymeric nanocomposite from each matrix in this study, as well as biopolymeric nanocomposites synthesized in previous literature works, and some conventional polymers. As shown in Figure 7a, Young’s modulus of P_9%_, and G_15%_ biopolymers are higher than that of petroleum-based synthetic polymers such as polyvinyl chloride (PVC), polyphenylene sulfide (PPS), polyimide (PI), polyetheretherketone (PEEK), polymethyl methacrylate/acrylic (PMMA), polyethylene terephthalate (PET), polysulfone (PSU), polycarbonate (PC), polyamide (PA), polyamide nylon 6 (PA6), polyvinylidene fluoride (PVDF), polypropylene (PP), commonly used in the fabrication of next-generation durable films [10]. Also, Young’s modulus of H_13.5%_ resembles that of most synthetic polymers. Compared with other biopolymers, Young’s modulus and tensile strength of the G_15%_, P_9%_, and H_13.5%_ biopolymeric nanocomposites are much higher than those of polyvinyl alcohol with pectin (PVA/PEC 1:1) [10], CN−RH RF + 15% CN, and CN−RW RF + 15% CN (rice starch reinforced with 15% nanocrystals, extracted from rice husk and straw, respectively) [25], 6% GPE/2% CH (residual garlic husk extract with chitosan) [52], and HPMC/10mM AuNPs (HPMC with gold nanoparticles) [48].

It is necessary to focus on adjusting the material’s brittleness, as it can be a disadvantage for the application of the film [10]. Therefore, assessing the film’s toughness or deformation energy density is pivotal. High mechanical strength in pure materials typically provides low toughness. However, adding nanostructures and increasing their dispersibility and compatibility can enhance material toughness without decreasing mechanical strength. Furthermore, this work confirms such a trend, as increasing the BCNCs in the continuous phase of the biopolymers (gelatin and HPMC) led to a considerable increase in the toughness of the biopolymeric nanocomposites, with a36.33% increase for G/BCNCs and a 99.54% increase for H/BCNCs. For pectin, the slight increase of 0.32% in toughness indicates lower compatibility between the matrix and BCNCs. It is noteworthy that the values of the mechanical properties did not present a significant difference (*p* < 0.05) for 15% BCNCs by mass in the gelatin matrix, 14% for pectin, and 13.5% for HPMC. Therefore, it is not advisable to add high contents of nanocrystals. Excessive insertion of nanocrystals can lead to excessive agglomeration of BCNCs in the biopolymer matrix, leading to an unbalanced stress distribution during the tensile test [40]. This behavior may be related to the self-agglomeration processes of BCNCs instead of percolation.

Figure 7b–e presents the combined effects of the independent variables (matrix and reinforcement mass percentage) on the tensile strength (Figure 7b), Young’s modulus (Figure 7c), elongation at break (Figure 7d), and toughness (Figure 7e) of the experimental data presented in Figure 6a–c. Subsequently, the final model is presented, according to Equations (3) for tensile strength, (4) for Young’s modulus, (5) for elongation at break, and (6) for toughness, to predict the responses for a given level of each factor. Positive coefficients show the positive effects of the factors on the responses, while negative signs indicate opposite effects on the responses. Multiple regression analysis applied to experimental data led us to select a quadratic model to analyze the mechanical properties, as it maximized the adjusted R^2^ and R^2^ values (Table A2). The R^2^ is close to unity with a value of 0.9572 for tensile strength, 0.9747 for Young’s modulus, 0.9724 for strain and break, and 0.9733 for toughness.

Tensile Strength (MPa) = −4.4064 × 10^5^ + 8470.8324x + 23.9424y − 40.7021x^2^ − 0.2095xy – 0.0411y^2^ (3)

Young’s Modulus (GPa) = 6534.5398 − 128.3255x − 4.5423y + 0.6301x^2^ + 0.0457xy − 0.003y^2^ (4)

Strain (%) = 4864.6535 − 91.2923x + 0.632y + 0.4283x^2^ − 0.0063xy + 0.0011y^2^ (5)

Toughness (×10^6^ J/m^3^) = −2.8021 × 10^5^ + 5479.1021x + 435.9748y − 26.7634x^2^ − 4.1565xy + 0.0342y^2^ (6)

where x (biopolymer matrix), y (% reinforcement), and xy (interaction between biopolymer matrix, and % reinforcement).

The type of biopolymer and BCNCs both contributed to the production of biopolymeric nanocomposites with high tensile strength, and in each matrix, BCNCs showed a positive effect. That resonates with their structural configuration, which limits molecular mobility in the network [5]. Therefore, gelatin-based nanocomposites showed higher tensile strength values. The combined effect of BCNCs with each matrix enhanced Young’s modulus. The value of this property was higher for pectin-based films. HPMC films, on the other hand, demonstrated greater deformation and presented excellent toughness. Thus, even though gelatin and pectin films are suitable for applications requiring higher strength, care must be taken to avoid reaching the maximum load, as ultimate stress corresponds to rupture stress. HPMC biopolymeric nanocomposites, however, are more suitable for application requiring good toughness in packaging.

## 4. Conclusions

The methodology for extracting the BCNCs was effective, as the average diameters and lengths were consistent with the literature. The mechanical strength properties show that the aspect ratio of the nanocrystals (6.67) was sufficient to achieve a percolation threshold that allows low filler content to provide an effective reinforcement and pronounced rigidity of the percolating cellulose network. It is worth remembering that nanocrystal orientation, organization, and morphology also interfere with the mechanical properties of nanostructured materials. The AFM analysis shows that the nanocrystals have a needle shape, and the surface micrograph corroborates that the BCNCs were uniformly dispersed in the gelatin, pectin, and HPMC matrices, even when presenting different functional groups. Since the BCNCs are negatively charged nanostructures, and pectin is an anionic polymer, the matrix/nanocrystal interaction force was likely weak, and the reinforcement phenomenon is primarily due to the high rigidity of the crystalline cellulose. For the gelatin and HPMC matrices (cationic, and neutral polymers, respectively), the nanocrystal/matrix interaction had a more significant effect. Based on the toughness values obtained, the calculated NC content at the percolation threshold—above 10, 9, and 8.5% by mass—is not ideal for the respective addition to the gelatin, pectin, and HPMC matrices. A higher percentage of NCs by mass does not significantly alter the mechanical properties. The biopolymeric nanocomposites based on HPMC presented better results based on the analyses, especially the H_8.5%_. Moreover, the nanocrystals were obtained from residual biomass, following the trends of the circular bioeconomy, which could reduce packaging production costs. The results obtained have significant implications for the ongoing efforts of the scientific community, serving as a baseline for further analyses on whether conventional plastics can be replaced by sustainable composites from an economic point of view, as such implementation is especially viable in the environmental context.

## Figures and Tables

**Table 1:** Composition of biopolymeric nanocomposite films.

		Biopolymers		
Acronyms	BCNCs (wt. % Biopolymer)	Gelatin (wt. %)	Pectin (wt. % m)	HPMC (wt. %)
G_0_	-	2.0	-	-
G_5%_	5.0	2.0	-	-
G_10%_	10	2.0	-	-
G_15%_	15	2.0	-	-
P_0_	-	-	2.0	-
P_4%_	4.0	-	2.0	-
P_9%_	9.0	-	2.0	-
P_14%_	14	-	2.0	-
H_0_	-	-	-	2.0
H_3.5%_	3.5	-	-	2.0
H_8.5%_	8.5	-	-	2.0
H_13.5%_	13.5	-	-	2.0

## Data Availability

The original contributions presented in this study are included in the article. Further inquiries can be directed to the corresponding author.

## Appendix A

**Table A1:** Film thickness.

Acronyms	Thickness (mm)
G_0_	0.031^a^ ± 0.004
G_5%_	0.033^a^ ± 0.0074
G_10%_	0.032^a^ ± 0.0056
G_15%_	0.041^a^ ± 0.0037
P_0_	0.018^a^ ± 0.0074
P_4%_	0.023^a^ ± 0.0048
P_9%_	0.015^a^ ± 0.0032
P_14%_	0.019^a^ ± 0.0044
H_0_	0.037^a^ ± 0.0054
H_3.5%_	0.027^a^ ± 0.0016
H_8.5%_	0.039^a^ ± 0.0037
H_13.5%_	0.027^a^ ± 0.0023

Different letters indicate a significant difference (*p* < 0.05).

**Table A2:** Model summary statistics.

Source	SD				R^2^				Adjusted R^2^				Remark
	TS	YM	S	T	TS	YM	S	T	TS	YM	S	T	
Linear	1.3245	4.8451	4.6254	3.7201	0.3396	0.7621	0.7295	0.6023	0.1612	0.6272	0.6800	0.5883	
Quadradi	0.4355	1.9285	1.8541	1.7761	0.9572	0.9747	0.9724	0.9733	0.9256	0.9469	0.9482	0.9468	Suggested

TS (tensile strength), YM (Young’s modulus), S (strain), T (toughness).
